# Fabrication of pore-filling cation-exchange membrane from waste polystyrene and Spunbond Meltblown Spunbond (SMS) non-woven polypropylene fabric as the substrate

**DOI:** 10.1038/s41598-024-56961-y

**Published:** 2024-03-16

**Authors:** Hadi Asgari, Farideh Ghavipanjeh, Mohammad Reza Sabour, Daryoush Emadzadeh

**Affiliations:** 1 Department of Civil Engineering, K.N.Toosi University of Technology, P.O. Box 1969764499, Tehran, Iran; 2 Energy Department, Materials and Energy Research Center, P.O. Box 3177983634, Karaj, Iran; 3 Department of Chemical and Biological Engineering, University of Ottawa, Ottawa, ON K1N 6N5 Canada

**Keywords:** Chemical engineering, Environmental chemistry

## Abstract

Commercial ion-exchange membranes are typically thick, possessing limited mechanical strength, and have relatively high fabrication costs. In this study, we utilize a three-layer polypropylene fabric known as Spunbond Meltblown Spunbond (SMS) as the substrate. This choice ensures that the resulting membrane exhibits high strength and low thickness. SMS substrates with various area densities, including 14.5, 15, 17, 20, 25, and 30 g/m^2^, were coated with different concentrations of waste polystyrene solution (ranging from 5 × 10^4^ to 9 × 10^4^ mg/l) before undergoing sulfonation using concentrated sulfuric acid. The physicochemical and mechanical properties of the membrane were characterized and compared with those of commercial Neosepta CMX and Nafion-117 cation-exchange membranes. Remarkably, the fabricated membrane exhibited good performance compared to commercial ones. The cation-exchange capacity (2.76 meq/g) and tensile strength (37.15 MPa) were higher, and the electrical resistance (3.603Ω) and the thickness (130 μm) were lower than the commercial membranes.

## Introduction

From the industrial revolution onwards, huge amounts of litter have been released into nature^1^. In recent years, the increase in the production of plastic and its widespread use has caused a rise in solid waste that requiring great attention^2^. Waste Polystyrene (WPS) comprises about 10% wt. of the worlds of plastic waste unloaded in deposited landfills, with only a negligible amount recycled into relatively effective plastics^3^. Polystyrene is a form of plastic that is extensively employed in numerous industries, such as packaging. It is widely used in the beverage and food industry and at this time in almost every aspect of our daily lives^2^. Sadly, the polystyrene's complex structure and high molecular mass make it undegradable and disposable, resulting in environmental problems since they can remain in water and soil for a long period^4^. These waste polystyrenes could be used to be converted into valuable for converting into invaluable materials for different applications^5^. Therefore, due to the reusability of waste polystyrene and its non-toxicity, this polymer is known as a eco-friendly and low-cost material^6^. In the last few years, due to water scarcity in various parts of the world, scientists have increasingly considered improving the ion-exchange capacity and mechanical robustness and using inexpensive materials to develop ion-exchange membranes^7^. Using waste polystyrene as a main component for synthesizing ion-exchange membranes (IEM) can be a new idea^8^. Ion-exchange membranes are divided into two main categories, anionic and cationic. Cation-exchange membranes (CEM) work as an electrolyte for transition cations from the anode to the cathode, as well as preparing an obstacle to the passage of anions between the electrodes. The opposite of this is true for anion-exchange membranes (AEM)^9^. Prevalent techniques used for polymer membrane fabrication consist of spin-coating, solution casting, and commercially scalable dip-coating^10^. However, fabricating an ionexchange membrane requires the usage of monomers or dissolution of the polymer in a solvent and sulfonating them^1^.

According to the available data, styrene-based membranes can be the primary type of homogeneous cation-exchange membranes. They are prepared by sulfonating styrene copolymers like styrene-ethylene butylene-styrene, followed by solvent casting to produce a thin membrane film^1^. The first limitation of this chemistry is that at high sulfonation grades, the polymer’s swelling can decrease the final membrane’s mechanical strength.

To date, few studies have been performed on the use of WPS to fabricate cation-exchange membranes. One of the available studies on this topic comes from 2018, where proton exchange membranes (without substrate) were fabricated by a mixture of waste polystyrene and petrol and compared with the commercial Ultrex membrane. Although the performance of fabricated membrane was slightly poorer (almost one percent), it could be an environmental friendly and cost-effective alternative to Ultrex membrane^11^. Another research has investigated the membrane made with waste polystyrene in two sulfonated and unsulfonated cases. In this case, a fixed amount of waste polystyrene was dissolved in methyl acetamide and dried in an incubator, and then its characteristics were examined in two sulfonated and unsulfonated states. The results showed that the sulfonation increased the cation-exchange capacity from 0.105 to 0.536 meq/l and ionic conductivity from 4.357 × 10^–7^ to 1.327 × 10^–6^ S/cm ^5^.

According to the literature, the use of waste polymers such as polystyrene in the cation-exchange membrane is not only cost-effective but also, with proper sulfonation, meliorates the cation-exchange capacity of the membrane. Another requirement of a suitable membrane is its mechanical properties, which can be increased by using an appropriate substrate^12^. In fact, the use of two different layers, which are separately responsible for the mechanical properties and the ions transport, facilitates the fabrication process and reduces the membrane price^13^. This can be achieved by coating waste polystyrene solution on a microporous substrate and then sulfonation. This method provides an appropriate platform for the fabrication of heterogeneous cation-exchange membranes^13^.

The choice of substrate in pore-filling membrane fabrication significantly affects the structural and physiochemical properties of the membrane^14^. This underscores the importance of exploring different substrates to optimize factors such as thickness, porosity and strength^15^. To the best of the authors’ knowledge, there is no existing literature on fabrication of cation-exchange membranes using polypropylene fabrics either of SMS (Spunbond Meltblown Spunbond) or meltblown (M) kinds as the substrate and sulfonated WPS as the active layer.

The SMS fabric comprises three layers of non-woven microfiber material: two outer layers made of spunbond (S) polypropylene and a central layer of meltblown (M) polypropylene. The M layer, recognised for its common use as the inner component of face masks, features finer filaments, typically ranging from 1 to 5 µm in diameter. The inclusion of the meltblown layer in the middle contributes to an enhanced porous structure in the SMS substrate, facilitating even coating and potentially influencing ion transport. The spunbond layers flanking the meltblown fabric provide overall strength and integrity to the material, rendering it suitable for applications that require resistance to tearing and puncturing^16^. Both fabrics offer high flexibility at low cost, play a pivotal role in providing mechanical support, act as separators, serve as carriers for coatings, and enhance the overall efficiency and performance of cation-exchange membranes. SMS fabric is widely used in hygiene and disposable medical products, with its weight (area density) typically ranging from 15 to 200 g/m^2^^17^. The non-woven substrate acted as the base onto which the ion-exchange material was applied or coated. Its porous structure plays a crucial role in improving the accessibility of the substrate to the ion-exchange material during coating. In the context of Cation-Exchange Membranes (CEMs), this material is commonly a polymer capable of selectively exchanging cations^18^.

In this study, a cation-exchange membrane was produced using polystyrene food packaging waste, with SMS fabric serving as the substrate. The appropriate concentration of the polymer solution and the effective area density/grammage of the substrate were systematically examined. The created membranes were then compared with commonly used cation-exchange membranes, namely, Noesepta CMX and Nafion-117.

## Materials and methods

### Materials

Concentrated sulfuric acid (98%), dichloromethane, silver sulphate, sodium chloride, barium chloride, hydrochloric acid, phenolphthalein, sodium hydroxide, potassium hydrogen phthalate, and ethanol were purchased from Merck. Waste polystyrene (WPS) was collected from a food container source, and SMS non-woven fabric of different area densities (14.5, 15, 17, 20, 25, and 30 g/m^2^) as the reinforcing material was obtained from the Baftineh Company (Iran).

### Determining the appropriate polymer concentration

Here, the appropriate concentration of the polymer (WPS) solution for the membrane fabrication was investigated. At first the waste polystyrene underwent thorough pretreatment, involving crushing, washing, and drying. It was then cut into small flakes (2–5 cm) and subjected to a meticulous cleaning process using ultrasonication in deionized water and ethanol. The final step included overnight drying at 75 °C in an oven, ensuring optimal condition for use in cation exchange membrane.

Homogeneous solutions of 5 × 10^4^, 6 × 10^4^, 7 × 10^4^, 8 × 10^4^, and 9 × 10^4^ mg/l of WPS in dichloromethane were prepared. The rectangular pieces (3 × 5 cm^2^) of SMS fabrics with area density of 17 g/m^2^ were dipped in the polymer solutions and sonicated for 10 min. The pieces were then removed and hung to dry. The dried samples were then immersed in the concentrated sulfuric acid containing AgSO_4_ as the catalyst with a ratio of catalyst-to-acid (0.1 mg/ml). The samples were placed in a shaker incubator at 50 °C and 100 rpm for 90 min. Finally, the samples were removed and washed with serial solutions of sulfuric acid from 80 to 40% and with plenty distilled water at the end in order to remove the excess acids. Then, various tests were performed on the samples, including; FT-IR, SEM, cation-exchange capacity, water uptake, proton conductivity, and tensile strength.

### Choosing the appropriate substrate

The SMS fabric with different area density of 14.5, 15, 17, 20, 25, and 30 g/m^2^ was investigated in order to obtain the suitable SMS substrate for the membrane fabrication. First, the SMS fabrics were cut into pieces of 15 cm^2^, then their thickness were measured by a vernier caliper under the same conditions. Each fabric was coated with WPS solution of 8 × 10^4^ mg/l and sulfonated with sulfuric acid containing AgSO_4_ with a ratio of catalyst-to-acid (0.1 mg/ml). The samples were kept in a shaker incubator under the same conditions of time (90 min), temperature (50 °C), shaker speed (100 rpm). Finally, the samples were removed and washed with serial solutions of sulfuric acid from 80 to 40% and with plenty distilled water at the end in order to remove the excess acids. Eventually the physicochemical and mechanical properties of the membrane were characterized and analyzed by FT-IR, SEM, cation-exchange capacity, water uptake, proton conductivity, and tensile strength.

### Membrane fabrication

**Figure 1:**
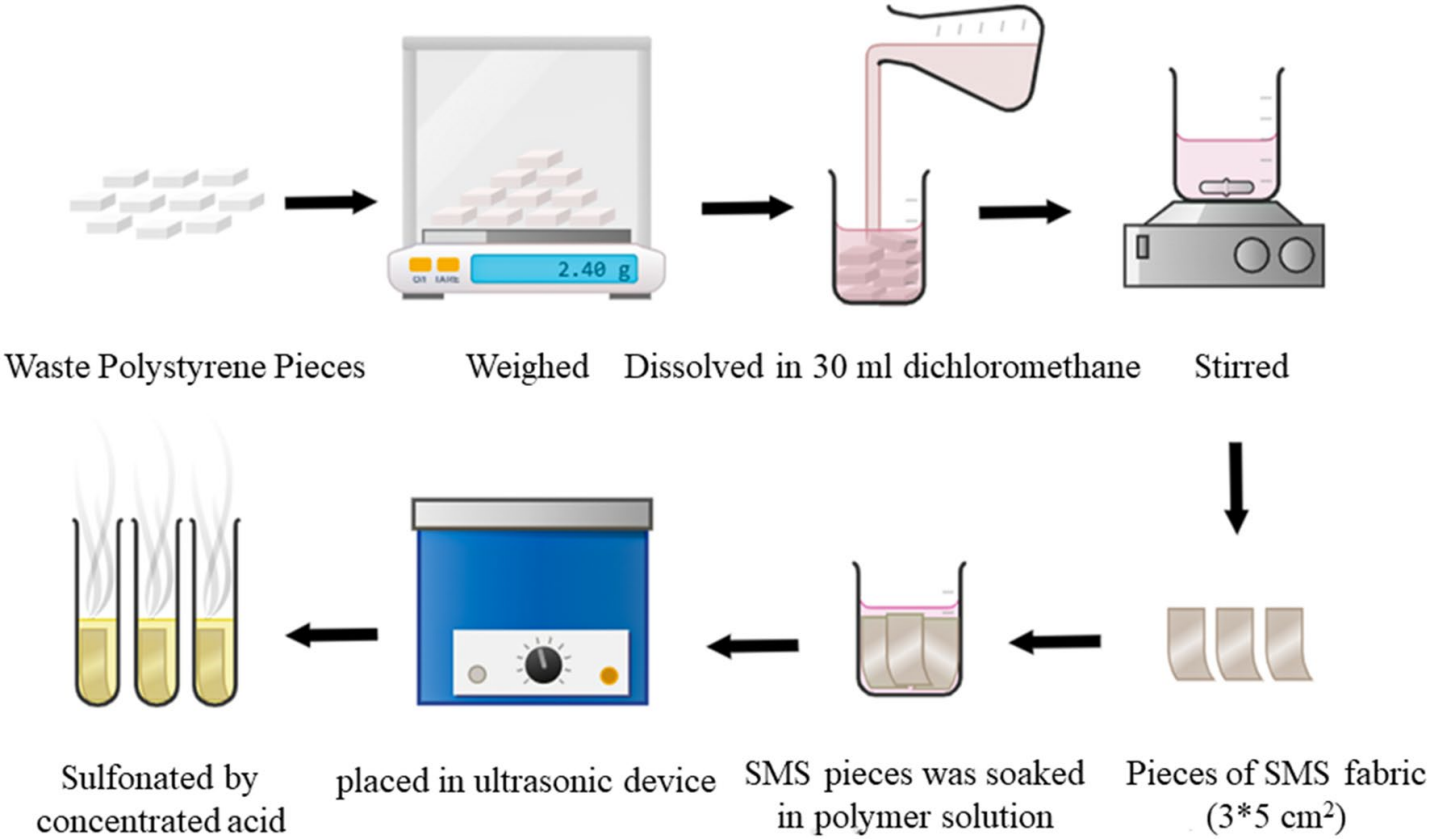
Schematic diagram of WPS membrane fabrication procedure.

A concentration of 8 × 10^4^ mg/l of dried WPS in dichloromethane was prepared in a sealed Erlenmeyer flask. The SMS fabrics with area density of 17 g/m^2^ were cut in dimensions of 3 × 5 cm^2^ and placed in the polystyrene solution and sonicated for 10 min. After that, the WPS-coated SMS fabrics were then hung at ambient temperature for 24 h to evaporate the solvent. The dried samples were then immersed in the concentrated sulfuric acid containing AgSO_4_ with a ratio of catalyst-to-acid (0.1 mg/ml). The samples were placed in a shaker incubator at 50 °C and 100 rpm for 90 min. Finally, the samples were removed and washed with serial solutions of sulfuric acid from 80 to 40% and with plenty distilled water at the end in order to remove the excess acids. Then the final product was examined with different tests including; FT-IR, SEM, cation-exchange capacity, water uptake, proton conductivity, tensile strength, and water permeability. The methodology of this study was depicted in Fig. 1.

### Membrane characterization; infrared spectroscopy

FT-IR spectral measurements were determined on a thermo scientific spectrophotometer (Nicolet iS10, USA) to confirm the fabricated membrane's chemical bonds and molecular structure. The samples were precisely mixed and ground with KBr salt to make the pellets and spectra were scanned in the range of the wave numbers 500–4000/cm.

### Membrane characterization; cation-exchange capacity

Cation-exchange capacity (CEC), defined as the quantity of positive exchangeable ions per unit mass of membrane, is used to measure the acidic group’s concentration of polymer electrolyte membranes. To remove the remaining acid, the sample membranes (3 × 5 cm^2^) were soaked in DI water for 24 h, and finally, their pH was measured to be sure that the acid has been completely washed off the membrane and the pH is within the neutral range^19^. The titration method at ambient conditions calculates the CEC of synthesized membranes. Pieces of 3 × 5 cm^2^ of each membrane were immersed in 30 mL NaCl solution (0.1 M) and agitated for 3 h on the shaker to convert hydrogen ions (H^+^) to sodium ions (Na^+^) and release them into the solution. The membrane was transferred to a beaker and the solution was titrated using NaOH solution (0.01 M) and phenolphthalein as indicators. The CEC value of membranes could be determined using Eq. (1)^20^:

1 $${\text{CEC }}\;\left( {\text{in milliequivalent}} \right)\; = { }\;\frac{{{\text{V}}_{{{\text{NaOH}}}} \times {\text{N}}_{{{\text{NaOH}}}} }}{{{\text{W}}_{{{\text{dry}}}} }}$$

where V_NaOH_ is volume of NaOH solution, N_NaOH_ is molarity of NaOH, W_dry_ is wight of dried membrane.

### Membrane characterization; scanning electron microscopic observation

The microscopic observations of the surface and cross-section of samples were carried out using JEOL JSM-6400, scanning electron microscope (SEM) device. For cross-section observation, the samples were first cooled in liquid nitrogen and then fractured.

### Membrane characterization; water uptake

Investigation of water absorbency of the fabricated membrane is essential to understand the desalination characteristics and ion transportation. The water uptake of the membrane was calculated using the mass of the wet and dry membranes. The membrane was cut into 3 × 5 cm^2^ and then placed in the oven at 60 °C for 3 h to dry completely. The sample was then cooled to room temperature in a desiccator and weighed (W_dry_). To determine the weight of the wet sample (W_wet_), it was immersed in deionized water for 24 h, then the sample was removed from the water, blotted to remove the excess amount of water and finally weighed. The water uptake capacity of the membranes was determined with the following (Eq. 2)^21,22^:

2 $${\text{Water }}\;{\text{Uptake }}\left( {\text{\% }} \right) = { }\frac{{{\text{W}}_{{{\text{wet}}}} - {\text{W}}_{{{\text{dry}}}} }}{{{\text{W}}_{{{\text{dry}}}} }} \times 100{\text{\% }}$$

where W_w_ and W_dry_ are the weight of dry and wet CEM (g), respectively.

### Membrane characterization; proton conductivity

The proton conductivity (σ) of the fabricated membrane was evaluated by Electrochemical Impedance Spectroscopy (EIS) method at a temperature of 25 °C with a potentiostate/galvanostate electrochemical device model MCTS-94A by AHNS CO. (Iran). A small piece (3 × 5 cm^2^) of the fabricated membrane was immersed in 30 ml NaCl solution (1.0 M) for 12 h^23^. Then the membrane sample was clamped between two Teflon blocks and fixed in place with plastic screws. Proton conductivity of the membranes was determined in frequency range of 0.1 Hz to 1 MHz at potential equal to 1 V across the electrodes. The following Eq. (3) were used to determine the proton conductivity of membranes^24^.

3 $${\upsigma } = \frac{{\text{L}}}{{{\text{R}} \times {\text{A}}}}$$

where, σ is the proton conductivity (S/cm), L is distance between two electrodes (cm), A is effective area (cm^2^), L/A is cell constant (1/cm), and R is electrical resistance (Ω).

### Membrane characterization; tensile strength

Tensile strength of the prepared membrane was measured using a uniaxial testing system SANTAM, STM20 at 25 °C and in 50% relative humidity, with a 100 N load cell and actuator speed of 5 mm/min^25^. The thickness, width, and length of the membrane were precisely measured using a vernier caliper and their values were estimated to be 130 μm, 5 and 3 cm, respectively^26^. Equation (4) was used to calculate the tensile strength^27^.

4 $${\text{T}} = \frac{{\text{F}}}{{{\text{t}} \times {\text{w}}}}$$

where T is tensile strength (MPa), F is the tensile force applied to membrane (N), t and w are thickness and width of membrane respectively (cm).

### Membrane characterization; water impermeability

Water permeability is a crucial parameter for assessing the performance of cation-exchange membranes and plays a fundamental role in their evaluation. This parameter signifies the volume of water passing through the membrane and serves as a key metric for measuring the efficiency and overall performance of the membrane. In ion-exchange membranes, an elevation in water flux can affect ion conductivity and may introduce other issues, necessitating precise adjustments in ingredient amounts to maintain a proper balance^28^. In this study, water permeability was assessed using a setup comprising an insulated Teflon chamber, a pressure gauge, and a peristaltic pump. The Teflon chamber, designed with two outlets and one inlet, featured one outlet in proximity to the membrane sample and another positioned beneath the sample and polypropylene granules. The circular fabricated membrane, with a diameter of 11 cm, was positioned within the Teflon chamber. To ensure membrane uniformity and prevent crumpling and indenting, the chamber was equipped with five layers of mesh netting with various hole sizes and polypropylene granules.

Following the standard for measuring water permeability (ISO/FDIS 7198), a 0.1 M NaCl solution was introduced into the system by applying a pressure of 16 kilopascals (equivalent to 0.16 bar) over 5 min. The flow was then calculated by determining the ratio of the volume of water passing through the membrane to time. Utilising the flow rate per effective membrane area and pump pressure, the water permeability was calculated in three repetitions, and the results were found to be consistent across all three tests. The water permeability of the membranes was accurately determined using this approach (Eq. 5).

5 $${\text{WP}} = { }\frac{{\text{V}}}{{{\text{A}} \times {\text{t}}}}$$

where V is the volume of solution that has passed through the membrane (ml), t is the time for solution to pass through the membrane (min), and A is the effective surface of the membrane (cm^2^).

**Figure 2:**
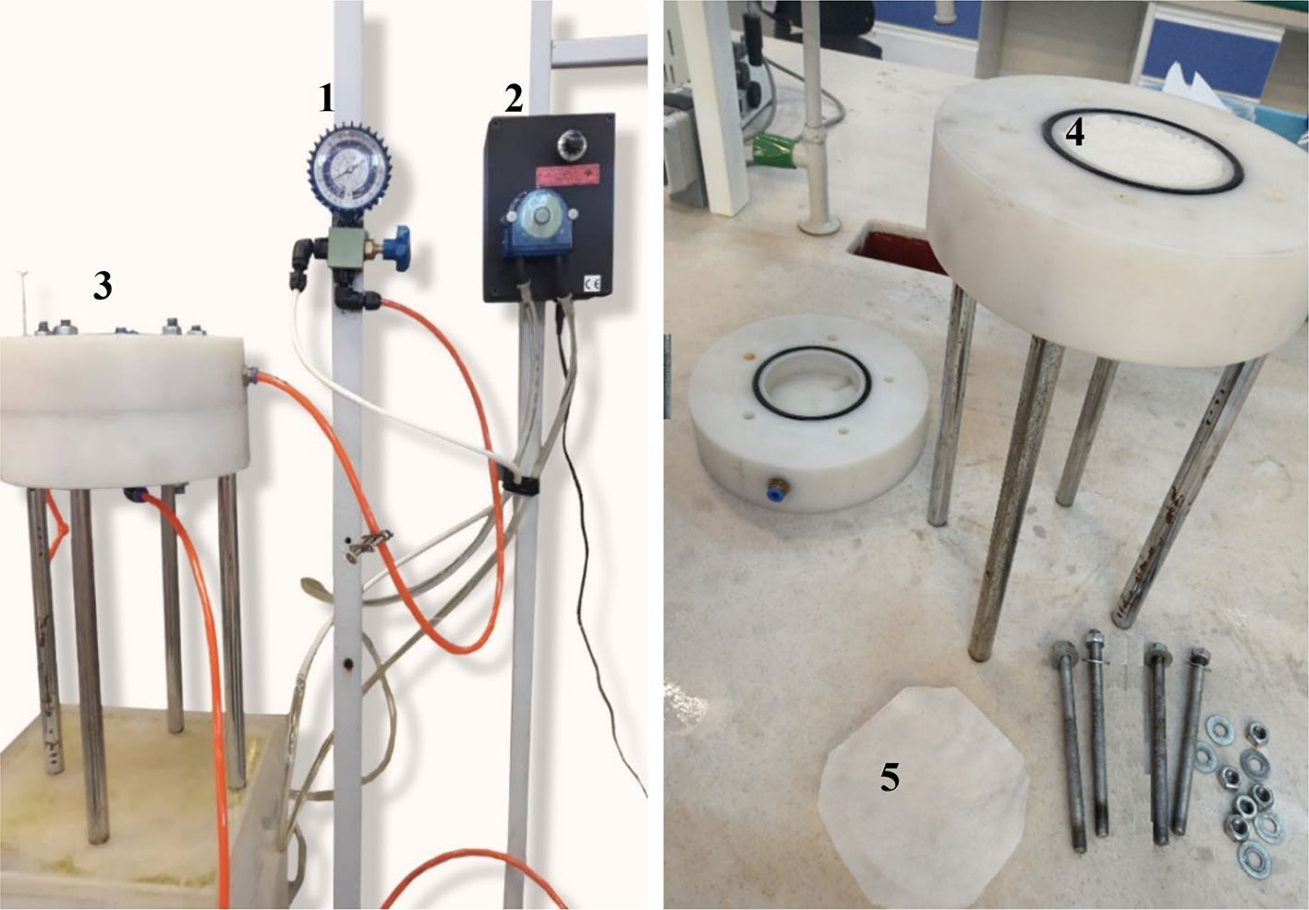
Schematic of the device for measuring the amount of passing NaCl solution. 1—Pressure gauge, 2—peristaltic pump, 3—Teflon water permeability system, 4—polypropylene granules, 5—mesh nets.

Figure 2 shows the laboratory pilot of water permeability test.

The membrane is characterized by FT-IR, SEM, cation-exchange capacity, water uptake, proton conductivity, contact angle, tensile strength, and water permeability tests and were compared with Neosepta CMX and Nafion-117 as commercial cation-exchange membranes.

## Results and discussion

### Determining the appropriate polymer concentration

The optimal concentration of the polystyrene solution is crucial for achieving a uniform and consistent coating on the membrane surface, which directly influences the thickness of the coated layer. By controlling the concentration, it is possible to optimize the coating thickness to attain the desired properties, including enhanced ion-exchange capacity and conductivity^29^. In this study, various concentrations of the polymer solution were applied to the substrate. Coating durations of less than 10 min resulted in a reduction in cation-exchange capacity, while durations exceeding this threshold led to fragility and a decrease in the mechanical strength of the membrane. After coating and measuring the thickness of the fabricated sample, its cation-exchange capacity was assessed.

**Figure 3:**
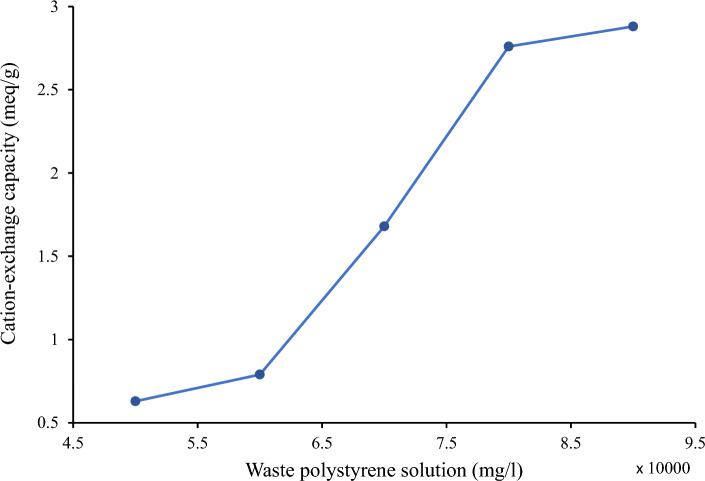
Cation-exchange capacities of the membranes made at different concentrations of waste polystyrene.

As shown in Fig. 3, the slope of the graph in the concentration of 6 × 10^4^ to 8 × 10^4^ mg/l of WPS solution has a significant increase compared to concentration of 5 × 10^4^ to 6 × 10^4^ mg/l. In addition, in the amount between 8 × 10^4^ and 9 × 10^4^ mg/l of polymer, the slope of the graph decreased compared to lower values. Even though, the maximum cation-exchange capacity was obtained as 2.88 meq/g at 9 × 10^4^ mg/l polymer solution, it is important to note that at this concentration, the membrane becomes fragile due to an increase in the thickness of the polymer on the substrate. In contrast, at the concentration of 8 × 10^4^ mg/l, the cation-exchange capacity of the membrane is nearly equivalent to that of the 9 × 10^4^ mg/l concentration, while also maintaining a more robust structure and a thinner profile. This lower thickness is advantageous for reducing electrical resistance. Therefore, 8 × 10^4^ mg/l was chosen as the appropriate polymer concentration for making the membrane in the next stages.

### Choosing the appropriate substrate

According to these findings, elevating the area density of the substrate to 17 g/m^2^ results in an increase in the cation-exchange capacity of the membrane, but beyond that point, the impact becomes insignificant. Striking the right balance between area density, cation-exchange capacity (CEC), ion conductivity, and thickness poses a key challenge in the production of effective cation-exchange membranes. While increasing the area density may contribute to a higher CEC owing to more available material for the attachment of ion-exchange groups, this relationship is not strictly linear, and diminishing returns may be observed as higher area densities are reached^30^.

The area density and thickness are closely intertwined, as the area density represents the weight of the fabric per unit area. Although thicker fabrics can offer advantages in terms of mechanical strength and durability, a trade-off exists. Excessive thickness may result in increased electrical resistance, posing a potential obstacle to ion conductivity. Balancing these factors is essential to ensure optimal membrane performance, considering both the mechanical and electrical properties of the material^21^. Therefore, the SMS fabric of area density 17 g/m^2^ was selected for fabrication of the membrane. Table 1 indicates the CECs of the membranes, thickness of the fabricated membrane, thickness of substrates, and also the amount of WPS loaded on each substrate based on grams per unit surface area of the substrate (3 × 5 cm^2^) with different area densities.

**Table 1:** The effect of the thickness and area density of the substrate on CEC.

Area density (g/m^2^)	Thickness of the fabric (mm)	Amount of loaded WPS (g/m^2^)	Thickness of the fabricated membrane (mm)	Cation-exchange capacity (meq/g)
14.5	0.05	16.80	0.09	1.86
15	0.06	17.94	0.11	2.21
17	0.07	19.42	0.13	2.76
20	0.09	22.84	0.16	2.81
25	0.12	24.97	0.16	2.83
30	0.14	27.74	0.22	2.90

### Membrane fabrication

The fabrication of a cation-exchange membrane involves critical decisions regarding the substrate, polymer solution concentration, coating time, catalyst-to-acid ratio in the sulfonation process, and duration and temperature of the sulfonation process. These parameters are pivotal, and values deviating from the optimal limits can lead to a significant decrease in various properties of the membrane. Careful control and optimization of these fabrication parameters are essential to ensure the desired performance and characteristics of the resulting cation-exchange membrane.

Here, the characteristics of the final membrane fabricated based on the selected area density of SMS fabric (17 g/cm^2^) and the selected WSP concentration (8 × 10^4^ mg/l) are presented.

### Membrane characterization; infrared spectroscopy

**Figure 4:**
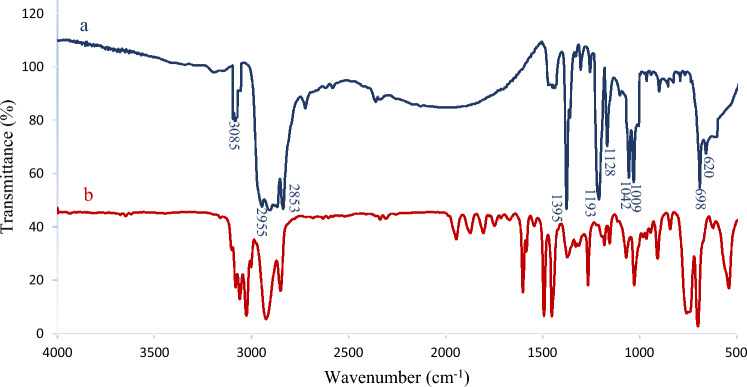
FT-IR spectra of (**a**) sulfonated waste polstyrene coated on substrate and (**b**) waste polystyrene.

Figure 4 shows the FT-IR spectra of the fabricated membrane. Among the notable peaks, a prominent one is observed at 3085/cm which are attributed to aromatic C–H stretch. Additionally, the characteristic peaks at 2955 and 2853/cm are assigned to asymmetric and symmetric CH_2_ vibrations, while the bands appearing at 1193 and 1128/cm correspond to asymmetric SO_2_ stretch^31,32^. Peaks at 1009, 1042/cm indicate symmetric SO_2_ stretch, and the presence of the (S–OH) group is verified by 698/cm^33^. These findings provide evidence of the sulfonation of waste polystyrene (WPS) filling the substrate’s pores. The sulfonation process using H_2_SO_4_ is a pivotal step in enhancing the functionality of cation-exchange membranes. This modification imparts unique properties to the membranes, improving their performance in various electrochemical applications. Sulfonation introduces sulfonic acid groups (–SO_3_H) into the polymer matrix, thereby creating ion-exchange sites. The negatively charged sulfonic acid groups attract and retain cations, enhancing the membrane’s efficiency in selectively allowing cations to pass through while impeding the movement of anions. Moreover, the chemical changes induced by sulfonation can lead to alterations in membrane surface characteristics, fostering a more porous structure. This modification influences the water uptake and membrane morphology, impacting the ion transport properties. The resulting membrane, with its sulfonated structure, exhibits improved ion-exchange capabilities and selective permeability, making it well suited for diverse applications in electrochemical systems^32^.

### Membrane characterization; cation-exchange capacity

The CEC of the membrane made with 8 × 10^4^ mg/l WPS solution on SMS fabric with an area density of 17 g/m^2^ at a sulfonation time of 90 min was equal to 2.76 meq/g. Notably, this CEC value of the Neosepta CMX membrane and Nafion-117 were reported as 1.64 meq/g and 0.93 meq/g respectively, while the CEC of fabricated membrane is considerably higher^34^. The observed significant difference in cation-exchange capacity (CEC) between the fabricated and commercial membranes can be attributed to several inherent factors. The thickness and overall structure of the membrane play a role in influence CEC. Thinner membranes facilitate faster ion transport. Additionally, the presence of a porous substrate and certain polymers, such as polystyrene in the fabricated membrane and perfluoro sulfonic acid polymers in Nafion-117, may affect the CEC. Optimal sulfonation conditions, including factors such as time, temperature, and catalyst-to-acid ratio, lead to distinct degrees of functionalization. A higher density of sulfonated groups in the membrane provides more active sites for cation exchange, contributing to increased CEC. The combination of these factors underscores the complexity of membrane fabrication and the importance of fine-tuning various parameters to achieve the desired ion-exchange properties^35^.

### Membrane characterization; scanning electron microscopic observation

**Figure 5:**
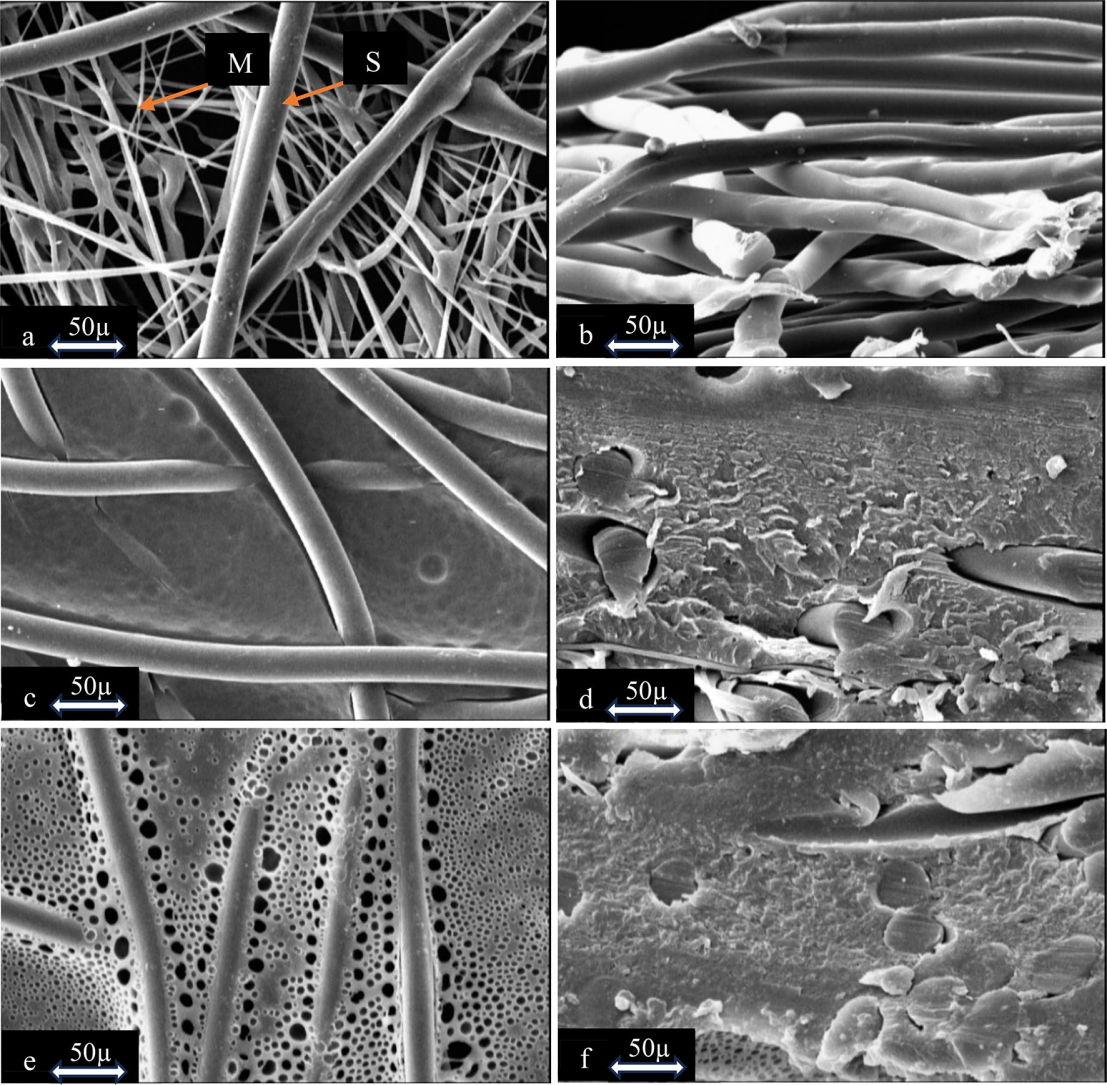
The SEM images of (**a**, **b**) the surface and the cross-section of the substrate, respectively, (**c**, **d**) the surface and the cross-section of the substrate after WPS coating, respectively, (**e**, **f**) the surface and the cross-section of the sample after sulfonation (the final membrane).

The SEM images in Fig. 5 depict the substrate and resulting cation-exchange membrane. In Fig. 5a, two types of filaments are visible: thicker spunbond and thinner meltblown fibres. Initially, both the meltblown and spunbond fabrics were individually coated. Although the meltblown fabric exhibited a relatively better cation-exchange capacity than the spunbond substrate, it suffered from an inadequately executed coating layer, displaying numerous holes and cracks on its surface and cross-section, along with very low mechanical strength. Consequently, using a combined substrate of these two fabric types (SMS non-woven fabric) addressed these weaknesses and improved the cation-exchange capacity. As evident in Fig. 5c and d, the WSP (presumably the coating material) effectively penetrated the substrate without any voids or gaps. Figure 5e shows a highly porous structure on the membrane surface, while such porosity is absent inside the membrane, as depicted in Fig. 5f. During the coating process, air bubbles infiltrated the empty spaces in the substrate. The application of ultrasound waves facilitates the transfer of these bubbles from the inner layers to the membrane surface. With the application of a relatively high temperature during the sulfonation process, these bubbles were eliminated, resulting in the formation of holes on the surface of the fabricated membrane^36^. This observation suggests that the polymer has been appropriately filled into the pores of the substrate during the membrane fabrication process.

### Membrane characterization; water uptake

The water uptake of the fabricated membrane was measured as 35.3%,whereas the water uptake of Neosepta CMX and Nafion-117 were reported as 29.9% and 17.4%, respectively^37,38^. Notably, the fabricated membrane has higher water uptake compared to that of the commercial Neosepta CMX and Nafion-117 membranes. The membrane’s structure, nature of functional group, and its porosity of the membrane all play significant roles in the interaction between the membrane and the water molecules. The SMS non-woven fabric, owing to the properties of its constituent materials, serves as a hydrophobic substrate. The meltblown fibres within the fabric were formed by melting a thermoplastic resin and extruding it through fine nozzles, creating a web of microfibres. This layer contributes to the hydrophobic nature of the SMS non-woven fabric, as many thermoplastic resins used in this process inherently repel water. Additionally, polystyrene, a synthetic polymer derived from the polymerisation of styrene monomers, is hydrophobic. Hydrophobic cation-exchange membranes may face challenges and limitations, such as issues with ion-exchange capacity and ion conductivity. Therefore, to enhance membrane performance, hydrophobicity is reduced through the sulfonation process. This process alters the hydrophobic nature of the membrane, addressing the challenges associated with ion-exchange capacity and ion conductivity. Striking a balance between the hydrophilicity and hydrophobicity of the membrane is crucial because it facilitates access to functional sites, resulting in an increase in the cation-exchange capacity and ionic conductivity^21,39^.

In contrast, a super hydrophilic property and high-water uptake, which may negatively impact membrane dimensional stability, can have a positive effect on membrane swelling. It is important to highlight that, while sulfonation can enhance the hydrophilicity of polystyrene, the specific conditions of the sulfonation process such as time, temperature, and the concentration of sulfuric acid will influence the degree of modification. Careful control of these parameters is necessary to achieve the desired hydrophilic properties without compromising the structural integrity of the polymer. Balancing these factors is crucial to ensuring both improved hydrophilicity and dimensional stability of the membrane^32^.

### Membrane characterization; ion conductivity

Measuring the ionic conductivity of membranes is important for evaluating the performance of membranes and the understanding of ion transport in them. Through the Electrochemical Impedance Spectroscopy (EIS) test, the electrical resistance (R) values and the cell constant (L/A) were obtained as 3.603 Ω and 0.5 1/cm, respectively. By substituting these values in Eq. (3), the value of proton conductivity was determined to be 0.138 S/cm. These results highlight that the proton conductivity of fabricated membrane is considerably higher than those of the Neosepta CMX membrane (0.055 S/cm) and Nafion-117 (0.035 S/cm)^38,40^. The electrical resistance of a cation-exchange membrane (CEM) is a critical parameter that significantly affects its performance in various applications. This resistance measures the extent to which the membrane hinders the flow of the electric current.

Several factors, including membrane thickness, water uptake, and ion-exchange capacity, influence the electrical resistance of the membrane. Generally, thicker membranes tend to have a higher resistance because ions must traverse a longer path through the material. The presence of water is essential for ion conductivity in CEMs because it facilitates the movement of ions within the membrane. Superhydrophobic membranes may exhibit higher electrical resistance, whereas excessive water uptake can lead to flooding and reduce the efficiency of ion transport. Achieving a balance between these factors is crucial for optimizing the electrical resistance of the membrane for its intended application^24^. A higher Ion-Exchange Capacity (IEC), which measures the number of ion-exchange groups per unit mass of the polymer, is a contributing factor that often leads to higher ionic conductivity. These variations in the parameters contribute to the differences in the electrical resistance and ionic conductivity among the membranes^41^. Therefore, the observed difference in lower electrical resistance and, consequently, greater ionic conductivity of the fabricated membrane can be attributed to its specific characteristics, such as thickness, water uptake, and cation-exchange capacity. For instance, the fabricated membrane has a thickness of 130 μm, a water uptake of 35.3%, and a cation-exchange capacity of 2.76 meq/l, compared to Neosepta CMX (170 μm, 29.9%, and 1.64 meq/l) and Nafion-117 (178 μm, 17.4%, and 0.93 meq/l).

### Membrane characterization; tensile strength

**Figure 6:**
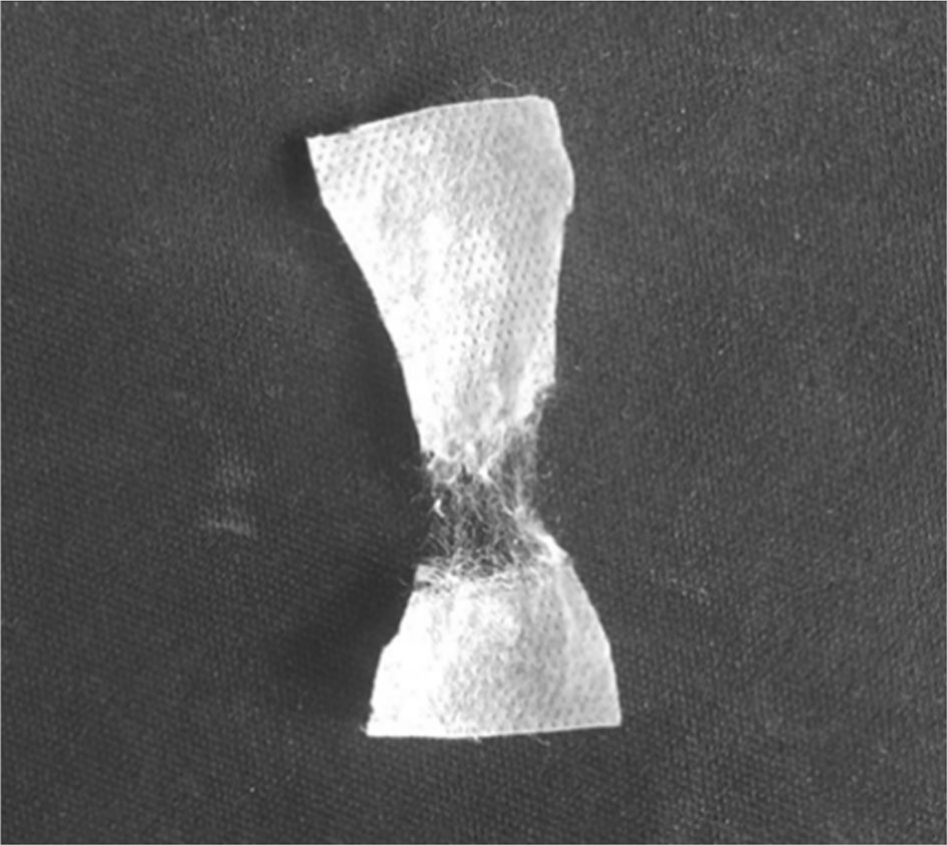
Rupture of the fabricated membrane (coated and sulfonated) after tensile strength test.

Figure 6 illustrates the fabricated membrane following the tensile strength test. As the force increases, the initial point of failure occurs as a localized rupture, subsequently leading to the formation of a hole in the membrane. Over time, this hole expands further, ultimately resulting in complete membrane rupture from that particular area.

Based on ISO 527, the appropriate temperature and humidity for testing the tensile strength of membranes are 25 °C and 50%, respectively, and increasing the temperature and humidity more than the mentioned values causes the tensile strength of the membrane to decrease. The tensile strength value for fabricated membrane and the commercial Neosepta CMX and Nafion-117 under the same conditions, was 37.15 MPa, 35.5 MPa and 24.5 MPa, respectively. The results reveal that the tensile strength of fabricated membrane is higher than that of Neosepta CMX and Nafion-117 membranes. This is due to the use of polypropylene substrate in the fabricated membrane, which increases the mechanical strength^37^. The type of polymer (polypropylene) used in the fabrication of the spunbonded and meltblown layers significantly affects the mechanical properties of the fabric. Thinner fibres of the meltblown layer generally contribute to increased flexibility, whereas thicker fibres of the spunbond layer provide strength and stability to the SMS non-woven fabric. Fibres in the spunbond layer are typically continuous and oriented in a specific direction, creating a durable and cohesive structure, and in the context of a cation-exchange membrane, these layers can act as a supportive base. Thus combination of these fibres, causes a balance between mechanical strength and other properties such as appropriate coating capability^16^.

### Membrane characterization; water permeability

The rate of passage of the salt solution through the membrane over 5 min, based on Eq. (4), resulted in a calculated water permeability of 438 ml/cm^2^ × min for the fabricated membrane. In comparison, the reported water permeabilities of Neosepta CMX and Nafion-117 were 391 ml/cm^2^ × min and 283 ml/cm^2^ × min, respectively. These values provide insights into the relative efficiency of water transport through the membranes, with the fabricated membrane exhibiting a higher water permeability than the commercial Neosepta CMX and Nafion-117 membranes^42,43^. The observed higher water permeability of the fabricated membrane compared to that of common commercial membranes aligns with the expectations from the water uptake test. Additionally, membrane morphology is a contributing factor to water permeability. Thicker membranes may inherently provide better resistance to water penetration. However, it is crucial to strike an optimal balance in choosing membrane thickness to ensure a harmonious combination of water impermeability with other performance factors. This emphasises the importance of carefully considering various parameters to achieve the desired membrane properties for specific applications^44^. SMS non-woven fabrics are frequently chosen for their durability and water-repellent properties, which can significantly contribute to the water impermeability of the membrane. These fabrics, comprising spunbond and meltblown layers with fine fibres in the micron- and nano-sized range, play a crucial role in creating a tortuous path and acting as a barrier against water penetration. The presence of these layers makes it more challenging for water to pass through the membrane, thereby neutralising the effect of the reduced membrane thickness. The combination of these factors enhances the water impermeability of the membrane and contributes to its overall performance and durability^45^.

## Conclusions

The cation-exchange membrane was successfully fabricated using a pore-filling membrane fabrication method based on waste polystyrene and SMS non-woven fabric as the substrate. The novelty of this research lies in the use of waste polystyrene on a cheap and firm polypropylene fabric (SMS fabric), which reduces the costs of the fabricated membrane. The SEM image shows that the waste polystyrene solution has penetrated effectively into the pores of the substrate due to coating, and a proper connection has been established between the solution and the substrate; the SEM of the cross-section of the membrane shows that the solution remains integrated with the substrate after sulfonation. The presence of sulfone groups, indicating the cation-exchange capacity of the fabricated membrane, was proved by the FT-IR results. The cation-exchange capacity test demonstrated that the fabricated membrane has perfect performance in adsorbing Na^+^ and releasing H^+^ when water passes through it. A tensile strength test (37.15 MPa) indicated that the use of SMS fabric as a membrane substrate improved the mechanical properties compared to the commercial samples. Other tests such as, ion conductivity (0.138 S/cm) were also performed, which indicate the better performance of the fabricated membrane compared to the Neosepta CMX and Nafion-117 membranes.

## Data Availability

The datasets used and analysed during the current study available from the corresponding author on reasonable request.
